# Metastatic Colorectal Cancer Presenting as Spinal Cord Compression and Mimicking Tuberculosis

**DOI:** 10.7759/cureus.83928

**Published:** 2025-05-11

**Authors:** Ahmed Amer, Ibrahim Abouelkhir, Muhammad O Kamal, Michael Shakhloul

**Affiliations:** 1 Emergency Medicine, Royal Surrey County Hospital, Guildford, GBR; 2 Emergency Medicine, Frimley Park Hospital, Frimley, GBR

**Keywords:** cancer of unknown primary, metastatic adenocarcinoma, spinal cord compression, tuberculosis mimicry, vertebral metastasis

## Abstract

A 69-year-old male presented to the ED with a three-week history of back pain and progressive bilateral lower limb weakness. Initial imaging raised concerns for discitis and spinal cord compression (SCC). Further investigations, including MRI, CT, PET-CT, and biopsy, revealed metastatic adenocarcinoma of likely lower gastrointestinal origin, complicated by pulmonary embolism and multiple pulmonary nodules. The case emphasizes the diagnostic challenges when malignancy mimics infectious processes such as tuberculosis and highlights the critical role of imaging, tissue biopsy, and multidisciplinary management in patients presenting with SCC.

## Introduction

Spinal cord compression (SCC) is a neurosurgical emergency often resulting from metastatic malignancy, but it can occasionally mimic infectious or inflammatory conditions. Early identification and intervention are paramount to preserve neurological function and improve outcomes [1]. This case discusses the evolving diagnostic journey of a 69-year-old male initially suspected to have spinal tuberculosis, ultimately diagnosed with metastatic adenocarcinoma, likely of colorectal origin, emphasizing the importance of maintaining a broad differential diagnosis and adopting a systematic multidisciplinary approach.

## Case presentation

A 69-year-old male, a professor of physics visiting his son in the United Kingdom, presented to the ED with a three-week history of severe lower back pain and progressive bilateral lower limb weakness. His symptoms had begun approximately five weeks earlier while in Nigeria, initially managed with physiotherapy, but the pain steadily worsened despite treatment.

Over the following weeks, he developed increasing difficulty with mobility, eventually requiring the use of a walking stick. After traveling to the United Kingdom, his condition deteriorated further, and within two weeks, he became chairbound, losing the ability to walk. In the days preceding his attendance, he developed urinary and fecal incontinence.

He also reported an unintended weight loss of approximately 10 kilograms during this time, accompanied by reduced appetite. He denied night sweats, cough, or hemoptysis. His past medical history was significant only for peptic ulcer disease, with no known exposure to tuberculosis or malignancy.

On examination, the patient was alert but chair-bound. Neurological assessment revealed complete loss of motor function in both lower limbs (0/5 power), absent anal tone, and loss of perianal sensation. Sensory examination demonstrated a level of impairment from the T9 dermatome downwards. His vital signs recorded a blood pressure of 145/90 mmHg, a heart rate of 119 bpm, and an oxygen saturation of 94% on room air. Abdominal examination revealed generalized tenderness without signs of peritonism.

Laboratory investigations showed a markedly elevated CRP of 184 mg/L and mild leukocytosis with a white blood cell count of 11.5 × 10⁹/L. Hemoglobin was within normal limits at 122 g/L. Liver function tests demonstrated a mild elevation in alanine transaminase at 66 U/L, while serum albumin was reduced at 30 g/L. Platelet count was within the normal range. All blood results are shown in Table 1.

**Table 1:** Blood results ALP, alkaline phosphatase; ALT, alanine transaminase; APTT, activated partial thromboplastin time; eGFR, estimated glomerular filtration rate; MCV, mean corpuscular volume; PT, prothrombin time; WCC, white cell count

Test	Result	Reference range
Sodium	140	133-146 mmol/L
Potassium	4.6	3.5-5.3 mmol/L
Urea	6.6	2.5-7.8 mmol/L
Creatinine	77	60-110 µmol/L
eGFR (EP)	88 (L)	>90 mL/min/1.73 m²
Albumin	30 (L)	35-50 g/L
Total bilirubin	12	<21 µmol/L
ALP	87	30-130 IU/L
ALT	66 (H)	<55 IU/L
Amylase	60	28-100 IU/L
CRP	184 (H)	<5 mg/L
IgG	19.5 (H)	7-16 g/L
IgA	2.84	0.7-4.0 g/L
IgM	1.01	0.4-2.3 g/L
Paraprotein	63	-
WCC	11.5 (H)	4.0-11.0 × 10⁹/L
Hemoglobin	122 (L)	130-180 g/L (M)
Platelets	338	150-400 × 10⁹/L
RBC	4.19 (L)	4.5-5.9 × 10¹²/L
Hematocrit	0.371	0.40-0.54
MCV	88.6	80-100 fL
Neutrophils	10.9 (H)	2.0-7.5 × 10⁹/L
Lymphocytes	0.9 (L)	1.0-3.0 × 10⁹/L
APTT	18.7 (L)	25-35 seconds
PT	16.4 (H)	11-14 seconds

**Figure 1:**
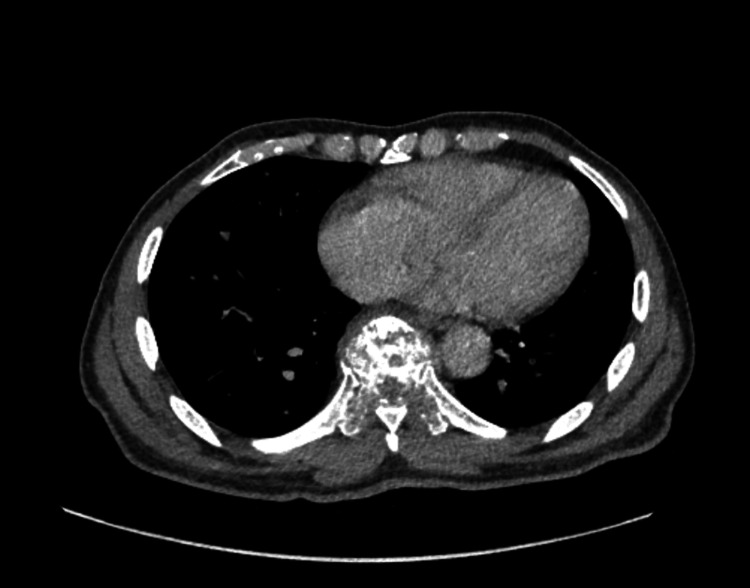
Contrast-enhanced CT scan of the abdomen and pelvis demonstrating lytic destruction of the T9 vertebral body with severe encroachment into the spinal canal

Imaging studies revealed significant findings. A CT scan of the abdomen and pelvis demonstrated bony destruction at the T9 vertebral level with severe spinal canal encroachment (Figure 1), raising concern for SCC. Multiple enlarged retroperitoneal lymph nodes, up to 12 mm in size, were identified. Several pulmonary nodules were also noted, raising the possibility of malignancy, although infectious causes could not be excluded.

**Figure 2:**
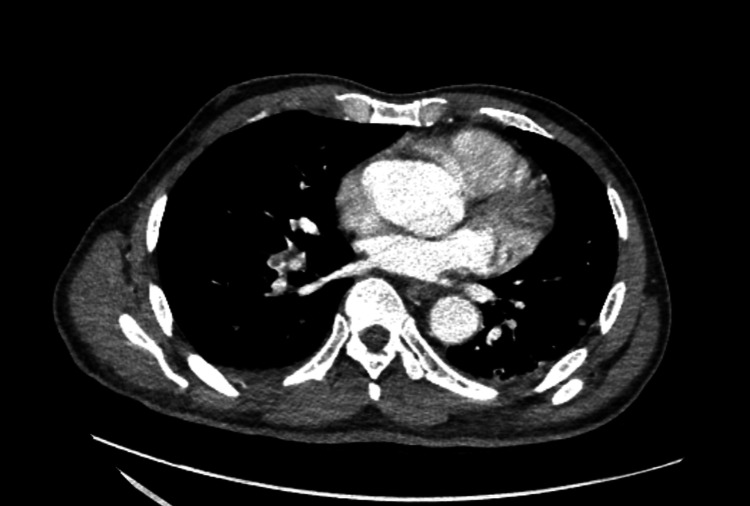
CT thorax showing bilateral mild-volume pulmonary emboli

A CT thorax further revealed bilateral mild-volume pulmonary emboli. Multiple pulmonary nodules were again visualized, consistent with possible metastases, alongside a small pleural effusion. Lytic destruction of the T9 vertebra, with involvement of the posterior elements and associated paraspinal and epidural soft tissue, was highly suggestive of a malignant process (Figure 2).

**Figure 3:**
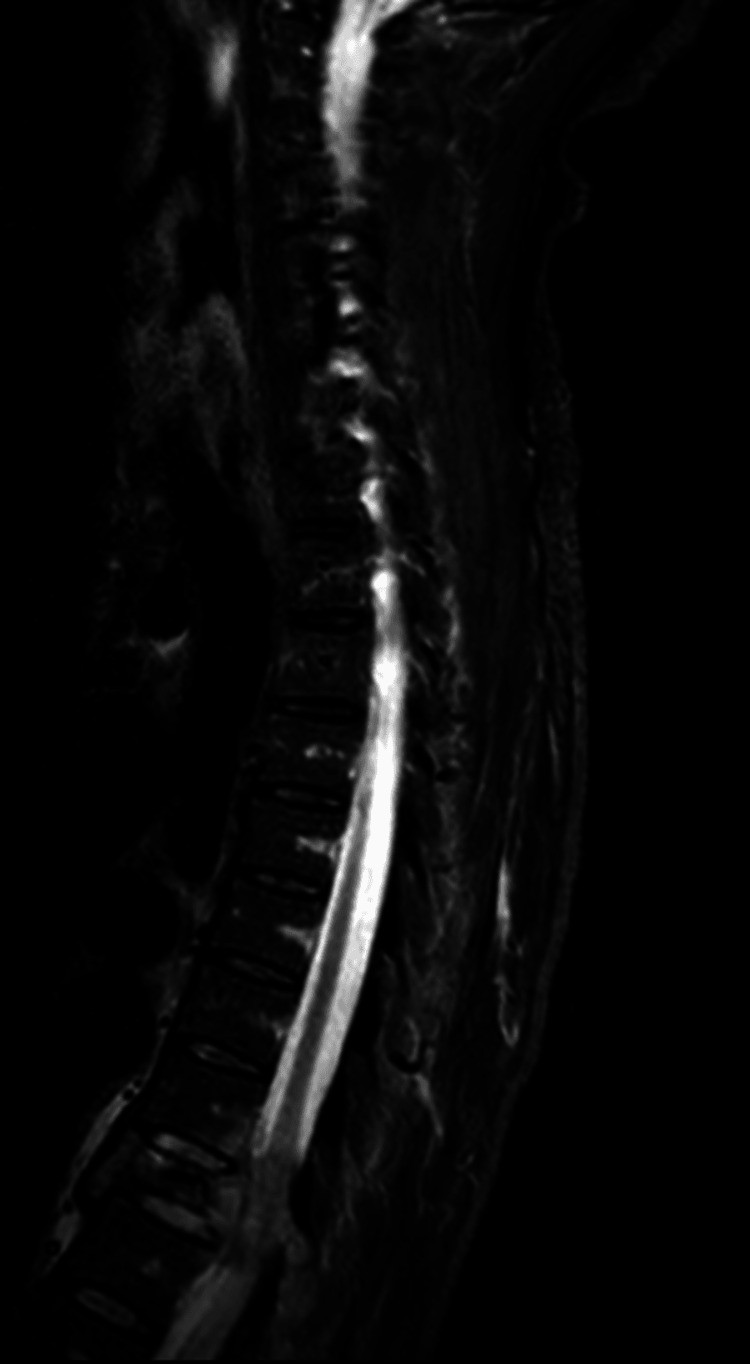
MRI of the thoracic spine showing a pathological fracture at T9 with an associated destructive soft tissue mass causing marked SCC SCC, spinal cord compression

An MRI of the spine demonstrated a pathological fracture at T9 associated with a large, destructive soft tissue lesion causing significant SCC. There was possible subligamentous spread into the adjacent T8 and T10 vertebrae, raising the differential diagnosis of tuberculous spondylitis; however, there were no associated epidural or paraspinal collections to support this diagnosis (Figure 3). Malignancy, particularly lymphoma or metastatic disease, was also strongly considered.

**Figure 4:**
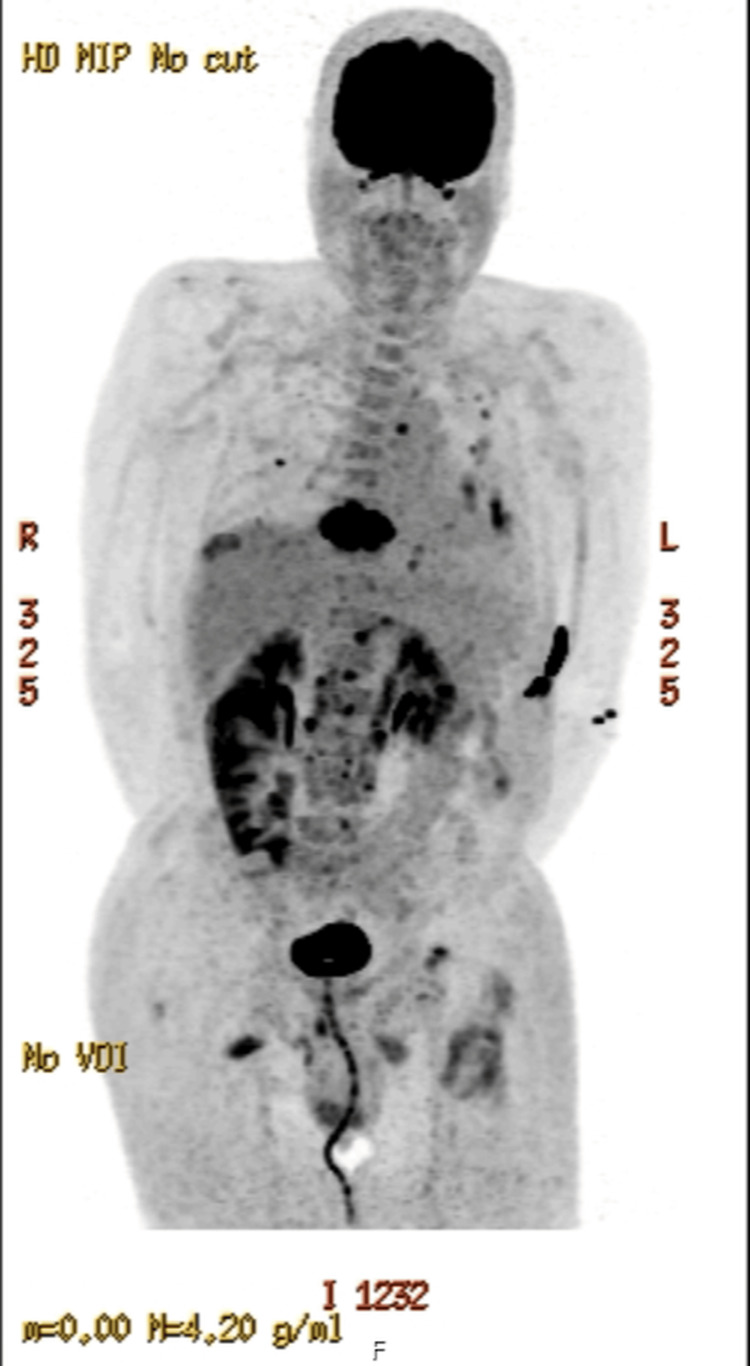
PET-CT scan demonstrating intensely FDG-avid collapse of the T9 vertebral body with an associated extraosseous soft tissue component, suggestive of malignant involvement FDG, fluorodeoxyglucose

A PET-CT scan revealed intense fluorodeoxyglucose (FDG) uptake in the collapsed T9 vertebra with an extraosseous component. Multiple FDG-avid pulmonary nodules with associated pleural effusions were present, along with hypermetabolic left lower paratracheal and upper abdominal lymphadenopathy (Figure 4). No definitive primary tumor was visualized.

The patient was commenced on intravenous flucloxacillin for presumed discitis and started on treatment-dose dalteparin for pulmonary embolism. Empirical treatment for disseminated spinal tuberculosis was initiated while awaiting histological confirmation. High-dose dexamethasone (8 mg twice daily) was administered for SCC.

**Figure 5:**
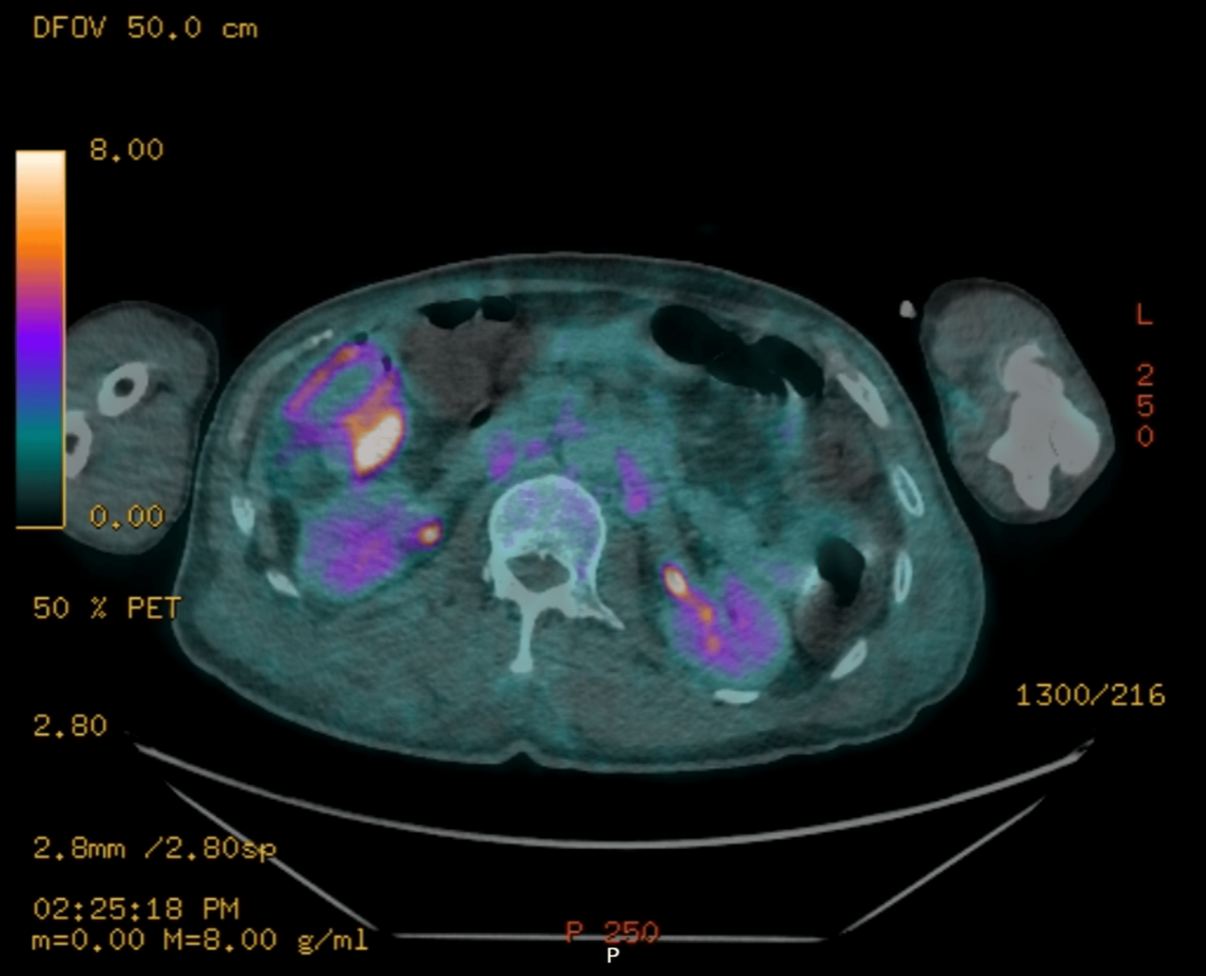
PET-CT showing a focus of FDG uptake in the distal ascending colon FDG, fluorodeoxyglucose

**Figure 6:**
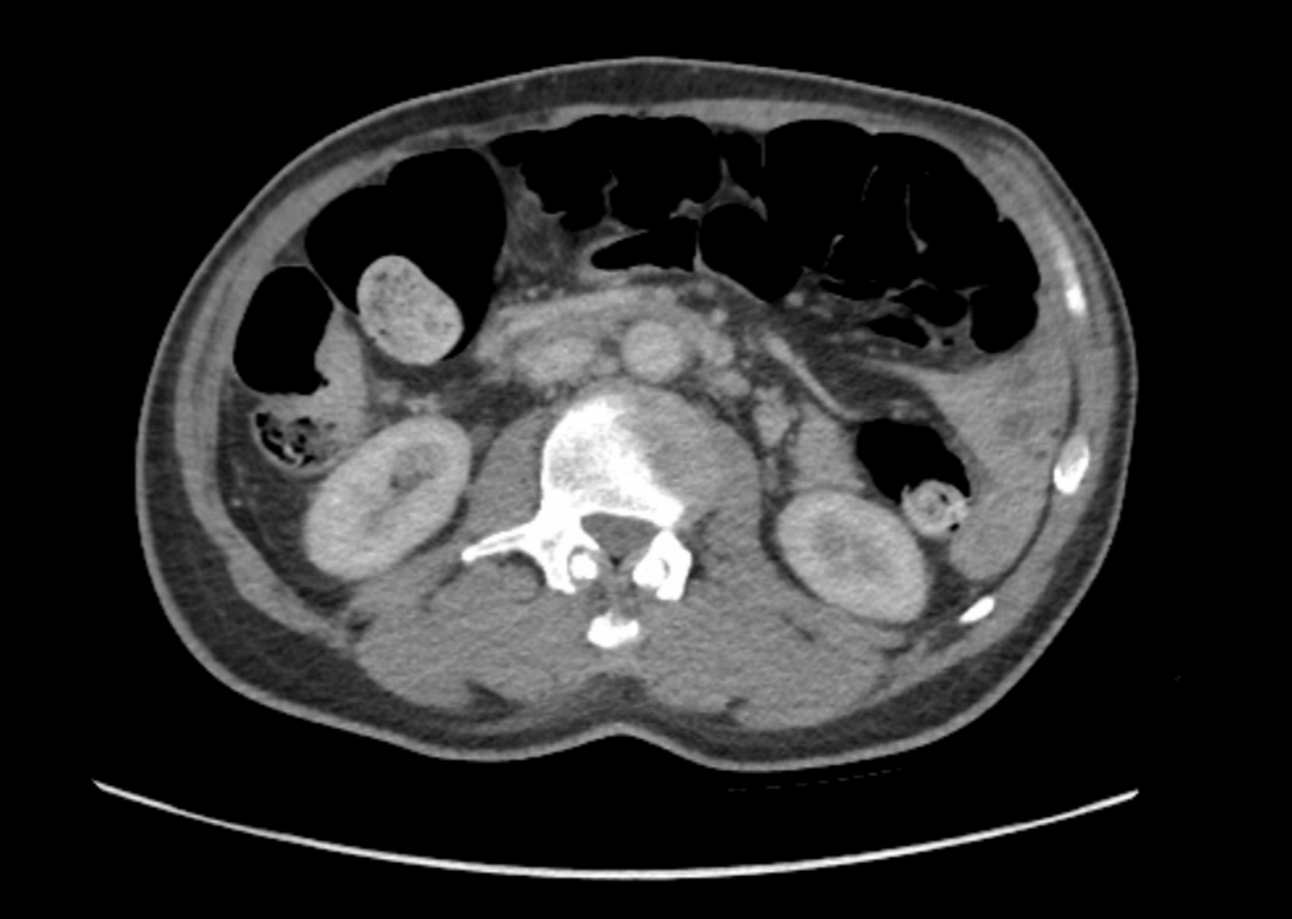
Abdominal CT showing focal wall thickening of the colon

Imaging was reviewed at the Cancer of Unknown Primary Multidisciplinary Team meeting. A review of imaging showed a focus of FDG uptake in the distal ascending colon on recent PET-CT (Figure 5). A review of a prior abdominal CT showed focal wall thickening of the colon in this region, together with infiltration of adjacent fat and small localized nodes consistent with the likely primary (Figure 6).

CT-guided biopsies of the T9 lesion subsequently confirmed the diagnosis of metastatic adenocarcinoma. Two biopsies showed cores of bone tissue extensively infiltrated by a moderately differentiated adenocarcinoma consisting of glandular structures with cribriform architecture and necrosis.

Multidisciplinary discussions involving oncology, neurosurgery, respiratory, and palliative care teams guided the management plan. Given the widespread metastatic disease and the absence of a definitive primary tumor, the case was classified as cancer of unknown primary (CUP). Palliative radiotherapy was initiated to manage SCC, and symptom control measures were prioritized following detailed family discussions regarding the goals of care.

## Discussion

SCC is a well-recognized complication of metastatic malignancy, occurring in approximately 3-5% of patients with cancer [2]. Presenting symptoms, including back pain, lower limb weakness, and sphincter dysfunction, necessitate urgent evaluation. Imaging, particularly MRI, remains the gold standard for assessing the extent of cord involvement [1].

In this case, the initial differential diagnosis included both infectious and malignant causes. The MRI findings of vertebral body destruction with paraspinal involvement without abscess formation made tuberculosis a possibility, especially given global epidemiological considerations. However, the lack of systemic infectious symptoms and the subsequent PET-CT findings favoring widespread metastases shifted the working diagnosis toward malignancy.

The diagnosis of CUP is established when metastatic cancer is present without an identifiable primary tumor after standard investigations. CUP represents approximately 3-5% of all cancer diagnoses and is typically associated with poor prognosis due to its aggressive nature [3]. Histopathological analysis in this patient revealed features consistent with adenocarcinoma, likely of colorectal origin based on immunohistochemical profiling, although no definite primary tumor was identified radiologically.

Management of metastatic SCC involves immediate corticosteroid administration to reduce edema [4], urgent radiation therapy [5] or surgical decompression when feasible [6], and systemic treatment if indicated. This patient’s poor functional status, widespread metastases, and poor prognosis made palliative radiotherapy the most appropriate option.

This case highlights the complexity of diagnosing metastatic disease in patients with nonspecific symptoms and the importance of maintaining a broad differential diagnosis, especially when initial findings may mimic infection. It also underlines the vital role of multidisciplinary team input in guiding both diagnostic and therapeutic pathways.

## Conclusions

This case underscores the critical need for a thorough and progressive diagnostic approach in patients presenting with neurological and systemic symptoms. A combination of early imaging, histological confirmation via biopsy, and multidisciplinary team collaboration was pivotal in reaching the diagnosis of metastatic adenocarcinoma. Despite initial concerns for infectious etiology, malignant disease was ultimately confirmed, altering the management focus towards palliative care. Clinicians must maintain a high index of suspicion for malignancy in cases of SCC, even in the presence of findings suggestive of infectious diseases such as tuberculosis.

## References

[1] Singleton JM (2025). Spinal cord compression. StatPearls [Internet].

[2] Robson P (2014). Metastatic spinal cord compression: a rare but important complication of cancer. Clin Med (Lond).

[3] Laprovitera N, Riefolo M, Ambrosini E, Klec C, Pichler M, Ferracin M (2021). Cancer of unknown primary: challenges and progress in clinical management. Cancers (Basel).

[4] Skeoch GD, Tobin MK, Khan S, Linninger AA, Mehta AI (2017). Corticosteroid treatment for metastatic spinal cord compression: a review. Global Spine J.

[5] Hershkovich O, Sakhnini M, Gara S, Caspi I, Lotan R (2022). Acute metastatic spinal cord compression: urgent surgery versus radiotherapy and treatment result prediction versus actual results. Curr Oncol.

[6] Meyer HS, Wagner A, Raufer A, Joerger AK, Gempt J, Meyer B (2022). Surgery in acute metastatic spinal cord compression: timing and functional outcome. Cancers (Basel).

